# Liver Resection With Extrahepatic Disease: A Population‐Based Analysis of Thoughtful Selection

**DOI:** 10.1002/jso.27944

**Published:** 2024-10-28

**Authors:** Kelly M. Mahuron, Matthew C. Hernandez, Paul Wong, Darrell Fan, Philip H. G. Ituarte, Mustafa Raoof, Gagandeep Singh, Yuman Fong, Laleh G. Melstrom

**Affiliations:** ^1^ Division of Surgical Oncology, Department of Surgery City of Hope National Medical Center Duarte California USA

**Keywords:** colorectal liver metastasis, extrahepatic disease, liver resection, patient selection

## Abstract

**Background:**

The oncologic benefit of liver resection for colorectal liver metastases (CRLM) in the setting of concurrent extrahepatic disease (EHD) is controversial. We performed a population‐based, cross‐sectional study to determine the practice patterns and overall survival (OS) of patients with CRLM + EHD who underwent liver resection.

**Methods:**

Patients with CRLM + EHD were identified using the California Cancer Registry from 2000 to 2019. Records were linked to the Office of Statewide Health Planning Inpatient Database. Patient demographics, clinical characteristics, and survival were compared between CRLM + EHD patients with and without liver resection.

**Results:**

Of 170 978 patients with CRLM, 62 003 (36%) had concurrent EHD (CRLM + EHD). In all, 3736 (6%) of CRLM + EHD underwent liver resection compared to 22% of patients with liver limited CRLM. Compared to CRLM + EHD without liver resection, CRLM + EHD with resection were younger, had fewer comorbidities, received higher frequencies of perioperative chemotherapy, and were more likely to have only a single site of EHD rather than multiple sites. Median OS was significantly higher for CRLM + EHD with resection compared to without (52 vs. 27 months, HR 0.46 [95% CI 0.44–0.47], *p* < 0.001). Regarding the location of EHD, this survival benefit was observed with liver resection for lung, peritoneal, intraabdominal lymph nodes, ovarian, and bone metastases.

**Conclusions:**

In a large population‐based setting, subsets of CRLM + EHD patients that undergo liver resection are associated with prolonged survival. These results support surgery with thoughtful patient selection to optimize survival outcomes in this population.

## 1 Introduction

Colorectal cancer (CRC) remains a significant global health burden [1], and nearly 20% of CRC patients have metastatic disease at diagnosis, while an additional 25% develop metastases during their disease course, most commonly to the liver [2, 3]. In addition to CRC liver metastases (CRLM), extrahepatic disease (EHD) may develop in other locations, including the lungs, peritoneum, periportal/retroperitoneal lymph nodes, and bone. The standard of care for resectable CRLM is hepatic resection along with perioperative chemotherapy [4]. However, as the presence of EHD is associated with poor prognosis and has previously served as a contraindication to liver resection, the utility of liver resection in the setting of EHD is controversial [5, 6].

The overall survival of patients with metastatic colorectal cancer continues to improve with the advent of newer therapies and complex multidisciplinary care [7]. Several studies have sought to address oncologic outcomes after liver resection in the context of EHD. The consensus has been that carefully selected CRLM patients with concurrent EHD (CRLM + EHD) may benefit from a margin‐negative hepatic resection and EHD resection with the caveat that recurrence is common [5, 8, 9]. Several factors prognostic of poor survival have been identified for these patients and have included the number and size of CRLM as well as the location of EHD [5, 9]. Unfavorable EHD sites have included non‐lung sites such as the peritoneum and portal/retroperitoneal lymph nodes, in addition to multifocal EHD. These factors were identified to help guide CRLM + EHD patient selection for surgery, as formal guidelines are not available and practice patterns are constantly evolving.

Additionally, the advent of liver‐directed therapies such as transarterial chemoembolization (TACE) and Y90 radioembolization has added to the armamentarium of treatments available to treat CRLM [10]. These options have been used for both palliation and as neoadjuvant therapy to facilitate hepatic resection [11]. However, their survival benefits and comparative efficacy to hepatic resection in the setting of EHD remain unknown.

Currently, the available data regarding patient selection and surgical outcomes for CRLM + EHD has been limited to single institution series. In this paper, we performed a large population‐based, cross‐sectional study to determine the real‐life practice patterns and overall survival (OS) of patients with CRLM + EHD who underwent liver resection.

## 2 Methods

### 2.1 Data

Patient data from the California Cancer Registry (CCR) was merged with the California Office of Statewide Health Planning and Development (OSHPD) discharge data to create a population‐based, retrospective study design of patients with CRC diagnosed from 2000 to 2019. The CCR is a statewide cancer registry that reports demographic, diagnostic, treatment, and survival information. It is one of the most comprehensive cancer registries in the United States as reporting for cancer care is mandatory in the state of California.

The OSHPD database contains discharge information from all general acute care and nonfederal facilities in California, including both cancer‐ and noncancer‐related admissions. Diagnoses and procedures for each record are coded according to the International Classification of Disease 9th Edition (ICD‐9) or 10th Edition (ICD‐10). For this study, CCR‐OSHPD linked records from 2000 to 2020 were used to capture records of patients with CRC metastatic to the liver and with additional sites of extrahepatic metastases. Human subjects review, and approval for use of the CCR‐OSHPD linked data was obtained from both institutional and state‐level institutional review boards.

### 2.2 Patients

Patients were identified according to the ICD‐0‐3 histology code for CRC (CRC) (8000–8152, 8154–8231, 8243–8245, 8250–8576, 8940–8950, 8980–8981). Site‐specific codes were also used to identify CRC (C18.0, C18.2–C18.9, C19.9, C20.9). Of note, patients with site‐specific codes for appendix (C18.1) were excluded. Our study included patients with histologically confirmed CRC who had a diagnosis of colorectal liver metastases (CRLM). Patients were determined to have concurrent EHD if the dates of the EHD were before or on the date of diagnosis of liver metastases. Patients with an unknown source of primary tumor, less than 18 years of age, or more than two separate malignancies were excluded.

### 2.3 Data Variables

The following variables were collected for analysis: age, sex, race/ethnicity, comorbidities based upon the Charlson‐Deyo score [12], primary colon site laterality, EHD site, resection of primary colon tumor, liver resection, and receipt of chemotherapy. Patient with liver metastases and additional sites of extrahepatic disease, including the lung, peritoneum, intraabdominal lymph nodes, ovary, adrenal gland, bone, and brain were identified using ICD‐9‐CM or ICD‐10‐CM diagnoses codes (Supporting Information S1: Table S1). Intra‐abdominal lymph nodes include retroperitoneal and porta hepatic lymph nodes [13] that have been previously associated with poor prognosis [5]. Liver resection, which included open or laparoscopic procedures, was also captured using ICD‐9‐CM and ICD‐10‐PCS procedures codes in Patient Discharge Data (PDD) files (Supporting Information S1: Table S2). Of note, open and laparoscopic ablation were also included as they are often combined with surgical resection for a parenchymal‐sparing approach to allow for repeat hepatic interventions as surgery for CRLM has a high rate of recurrence [14].

### 2.4 Statistical Analysis

Student's *t* test was used for continuous variables, while the *χ* ^2^ test was used for categorical variables. Survival curves were compared using the Kaplan–Meier method and log‐rank test. Follow‐up was defined as the time from the date of diagnosis to the date of death or last contact. For overall survival, a failure event was defined as any death. Univariate analysis was performed using the Cox proportional hazards model. Statistical significance was set at *p* < 0.05. Statistical analysis was performed using Stata MP 14.2 software (StataCorp LLC, College Station, TX).

## 3 Results

**Figure 1:**
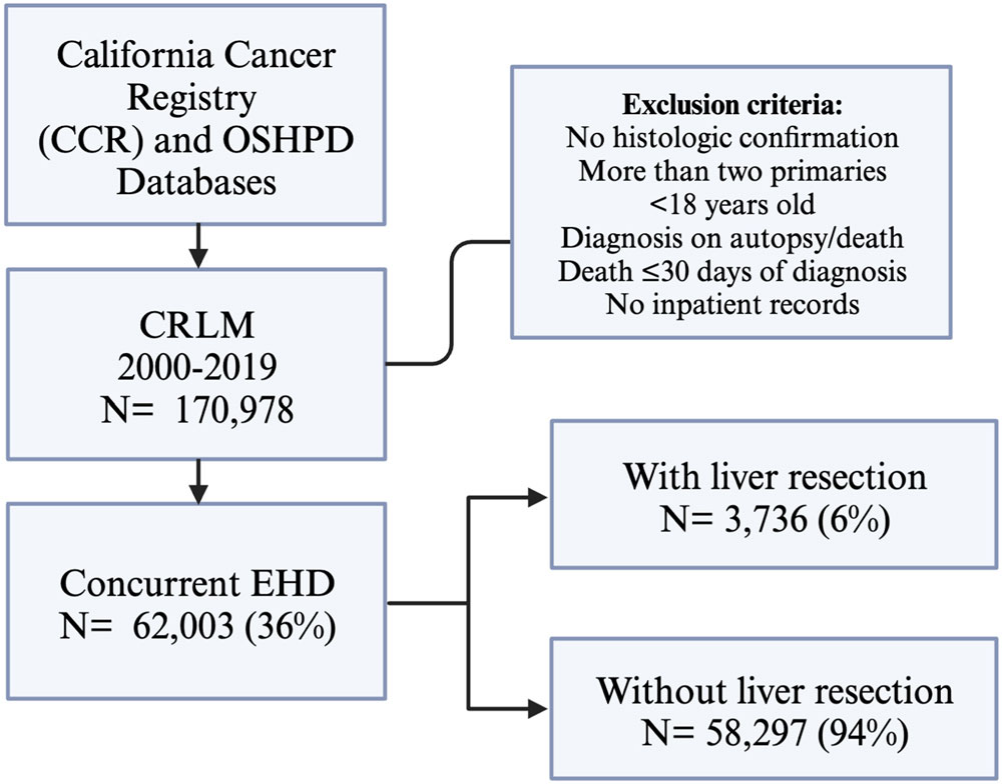
Study design and inclusion/exclusion criteria. CRLM, colorectal liver metastases; EHD, extrahepatic disease; OSHPD, California Office of Statewide Health Planning and Development.

Of the 170,978 patients with CRLM, 62,003 (36%) had concurrent EHD (CRLM + EHD). Overall, 16% of all patients with CRLM underwent liver resection. Interestingly, only 3736 (6%) of CRLM + EHD underwent liver resection (Figure 1) versus 22% of patients with liver‐limited CRLM.

Patient demographics, clinical characteristics, and treatments regimens for patients are shown in Table 1. Compared to patients with CRLM + EHD who did not undergo liver resection, CRLM + EHD patients who underwent resection were younger, had fewer comorbidities, and received higher rates of perioperative chemotherapy. Patients with resection were also less likely to be of minority race/ethnicity. Additionally, patients who underwent resection tended to have left‐sided primary colorectal tumors and higher rates of primary tumor resection. The most common sites of EHD in the liver resection group were the peritoneum (59%), intraabdominal lymph nodes (42%), lung (34%), and ovary (15%). The most common sites of EHD in the without liver resection group were lung (56%), peritoneum (43%), intraabdominal lymph nodes (34%), and bone (19%). Compared to patients without liver resection, CRLM + EHD patients with liver resection were more likely to only have a single site rather than multiple sites of EHD (88% vs. 73%, *p* < 0.001).

**Table 1:** Patient demographics and clinical characteristics by liver resection group.

Variable^a^	CRLM (*n* = 170,978)	CRLM + EHD without liver resection (*n* = 58,621)	CRLM + EHD with liver resection (*n* = 3382)	*p*‐value
Age (years), median (range)	62 (18–102)	61 (18–99)	56 (19–89)	< 0.001
Gender				0.003
Male	94 817 (55)	31 152 (53)	1700 (50)	
Female	76 148 (44)	27 457 (47)	1682 (50)	
Race/ethnicity				< 0.001
White	89 425 (52)	29 079 (50)	1980 (59)	
Black	15 638 (9)	5659 (10)	165 (5)	
Hispanic	35 739 (21)	12 615 (22)	526 (16)	
Asian/PI	24 365 (14)	9427(16)	580 (17)	
Other	616 (1)	616 (1)	25 (1)	
Comorbidities				< 0.001
None	39 230 (67)	2894 (78)	
One	26 954 (21)	10 589 (18)	546 (15)	
≥ Two	23 826 (18)	8488 (15)	256 (7)	
Primary site				< 0.001
Right colon	57 892 (35)	20 304 (36)	1063 (32)	
Left colon/rectum	107 587 (65)	35 982 (64)	2256 (68)	
Primary tumor resection				< 0.001
No	45 096 (26)	16 639 (28)	217(6)	
Yes	125 870 (74)	41 975 (71)	3165 (94)	
Concurrent EHD site^b^				
Lung	34 078 (20)	32 943 (56)	1135 (34)	< 0.001
Peritoneum	27 466 (16)	25 472 (43)	1994 (59)	< 0.001
Lymph nodes^c^	32 474 (19)	19 807 (34)	1410 (42)	< 0.001
Ovary	4106 (2)	3606 (6)	500 (15)	< 0.001
Bone	11 181 (7)	11 011 (19)	170 (5)	< 0.001
Adrenal	2177 (1)	2104 (4)	73 (2)	< 0.001
Brain	3379 (2)	3375 (6)	—d (< 1)	< 0.001
Number of EHD sites				< 0.001
0	108 975 (64)	—	—	
1	45 518 (27)	42 557 (73)	2961 (88)	
2	13 056 (8)	12 689 (22)	367 (11)	
≥ 3	3399 (2)	3375 (6)	54 (2)	
Any chemotherapy				< 0.001
No	51 377 (30)	17 727 (30)	655 (19)	
Yes	115 024 (67)	39 328 (67)	2710 (80)	
Overall survival				< 0.001
(months), median (95% CI)	31 (30–31)	27 (26–27)	52 (51–54)	

Abbreviations: CI, confidence interval; CRLM, colorectal liver metastases; EHD, extrahepatic disease.

^a^ Variables reported as *n* (%) unless otherwise specified.

^b^ Total not equal to 100% when multiple sites of EHD present.

^c^ Intraabdominal (including retroperitoneal/porta hepatitis) lymph nodes.

^d^ Denotes fewer than 15 cases.

**Figure 2:**
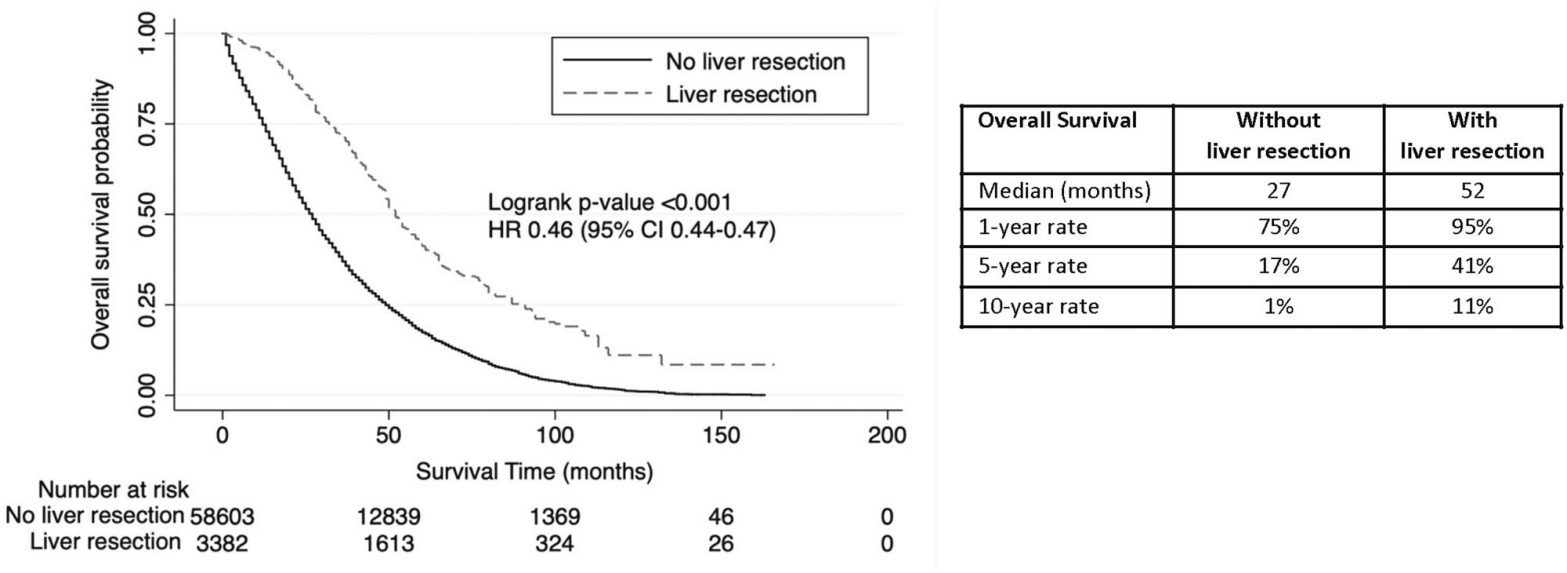
Kaplan–Meier survival analysis showing overall survival in patients with colorectal liver metastases and extrahepatic disease stratified by whether they underwent liver resection.

Compared to CRLM + EHD without liver resection, median OS was significantly higher with liver resection (52 vs. 27 months, HR 0.46 [95% CI 0.44–0.47], *p* < 0.001) (Table 1, Figure 2). When the site of EHD was considered, CRLM patients with lung, peritoneal, intraabdominal lymph nodes, ovarian, and bone metastases had significantly longer median OS when liver resection was performed (Table 2). The 1‐, 5‐, and 10‐year rates of OS with and without resection were 95% versus 75%, 41% versus 17%, and 11% versus 1%, respectively (Figure 2).

**Table 2:** Hazard ratio for death by site of extrahepatic disease and liver resection.

EHD site	Liver resection	Median OS (months)	HR (95% CI)	*p*‐value
Lung	Yes	61	0.39 (0.37–0.42)	**< 0.001**
	No	29		
Peritoneum	Yes	50	0.45 (0.45–0.48)	**< 0.001**
	No	24		
Lymph nodes^a^	Yes	54	0.48 (0.46–0.50)	**< 0.001**
	No	30		
Ovary	Yes	60	0.57 (0.51–0.63)	**< 0.001**
	No	33		
Adrenal Gland	Yes	50	0.79 (0.61–1.03)	0.0711
	No	30		
Bone	Yes	47	0.57 (0.48–0.67)	**< 0.001**
	No	33		
Brain	Yes	41	0.98 (0.69–1.38)	0.863
	No	33		

*Note:* The bold values are statistically significance.

Abbreviations: CI, confidence interval; EHD, extrahepatic disease; HR, hazard ratio; OS, overall survival.

^a^ Intraabdominal (including retroperitoneal/porta hepatitis) lymph nodes.

**Figure 3:**
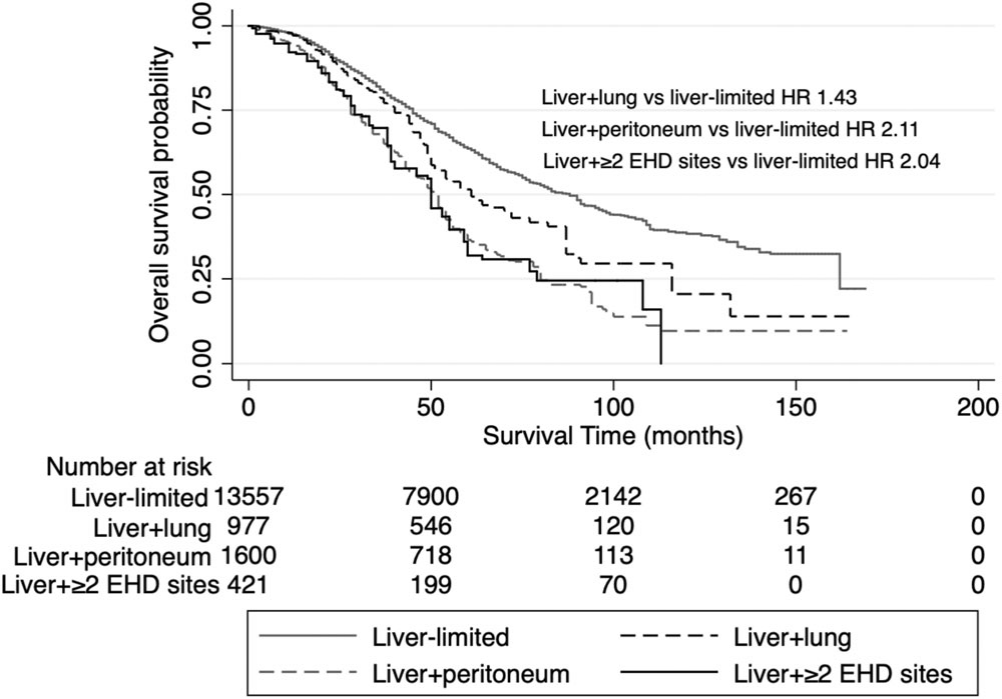
Kaplan–Meier survival analysis showing overall survival in patients with colorectal liver metastases who underwent hepatectomy stratified by whether they have (a) liver‐limited disease (no EHD), (b) lung‐only EHD, (c) peritoneum‐only EHD, or (d) multiple (2 or more) sites of EHD.

Survival curves comparing patients who underwent liver resection without EHD (liver‐limited), with liver and lung metastases, with liver and peritoneal metastases, and liver with multiple sites of EHD are shown in Figure 3. Median OS for the four groups was 88, 61, 52, and 50 months, respectively.

Among those with CRLM + EHD who underwent hepatectomy, there were 82 patients who survived 10 years or more (not shown). These patients had only a single site of EHD; 34 patients (41%) had lung metastases, and 48 patients (59%) had peritoneal metastases.

**Figure 4:**
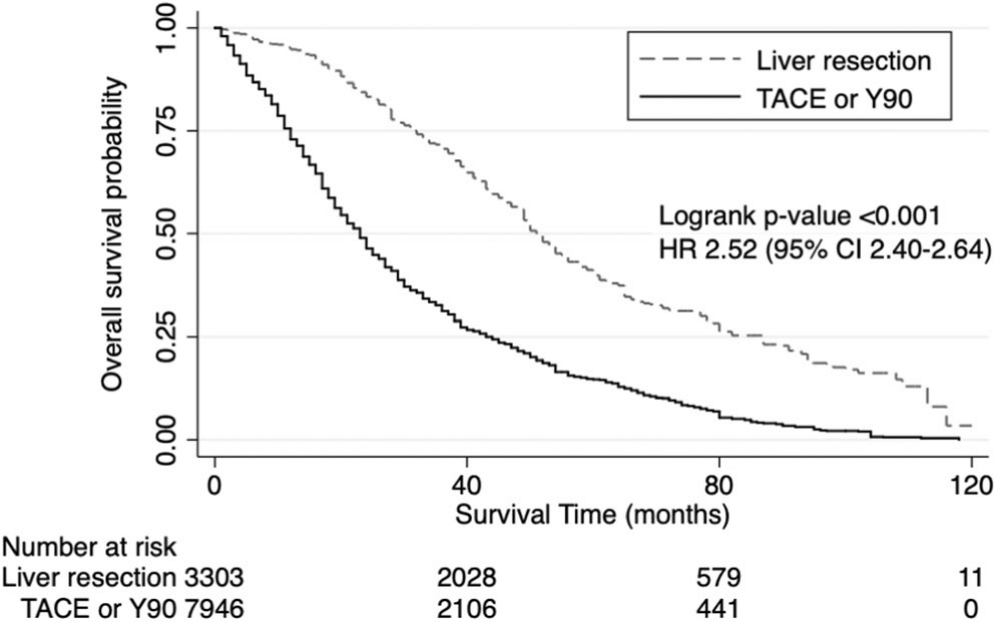
Kaplan–Meier survival analysis showing overall survival in patients with colorectal liver metastases and extrahepatic disease stratified by whether they underwent liver resection or liver‐directed therapy, including transarterial chemoembolization (TACE) or yttrium‐90 radioembolization (Y90).

In addition to liver resection, 7971 patients with CRLM + EHD underwent liver‐directed therapies, including transarterial chemoembolization (TACE) and Yttrium‐90 embolization (Y90) without resection. As shown in Figure 4, median OS was significantly lower in CRLM + EHD patients who underwent TACE or Y90 without resection compared to liver resection (23 vs. 52 months, HR 2.52 (95% CI 2.40–2.64), *p* < 0.001).

## 4 Discussion

This population‐based, cross‐sectional study describes current, real‐world practices involving liver resection for CRLM + EHD patients. In this study, we demonstrate that prolonged survival can be achieved for select CRLM + EHD patients undergoing hepatectomy.

Compared to CRLM + EHD patients who did not undergo liver resection, those who had liver resection tended to be younger with fewer comorbidities, supporting an expected bias towards surgical selection of fitter patients. This is also reflected in the high rates of perioperative chemotherapy (80%) in the resection group.

Regarding the location of EHD, the most common sites for the liver resection group were the peritoneum, intraabdominal/retroperitoneal lymph nodes, lung, and ovary. Less than 5% of the resection group had bone or brain metastases. This contrasts with the CRLM + EHD without resection group, which had higher rates of lung involvement as well as bone and brain metastases. Additionally, CRLM + EHD patients who underwent resection were more likely to have single rather than multiple sites of EHD compared to patients without resection (88% vs. 73%). These findings potentially reflect thoughtful selection of patients based on established data by select high‐volume centers which reveal worse long‐term outcomes for select groups of patients, particularly those with bone and brain metastasis [15].

We show that with liver resection, CRLM + EHD patients have a median OS of 52 months and a 5‐year survival rate of 41%. These survival statistics are significantly higher than for our no resection group (27 months, 17%), and they are comparable or higher than other studies of CRLM + EHD outcomes with hepatectomy in the literature [16]. As expected, the overall survival of the CRLM + EHD resection group is lower than the survival after hepatectomy for liver‐limited disease in this database (median OS 88 months, 5‐year survival rate 64%) and in other studies [17]. However, the survival of the CRLM + EHD resection group is still notably higher than for metastatic CRC patients receiving chemotherapy alone. Even with the recent advances in chemotherapy regimens, limited survival up to 30 months is reported in highly selected patients with unresectable disease [18].

Similar to other studies, the location of EHD impacted the survival benefit of liver resection in our study, with CRLM patients with lung metastases deriving the most benefit. This better prognosis with lung EHD compared to other sites has also been demonstrated in multiple other studies [5, 8, 19, 20]. Interestingly, in our study, the frequency of lung EHD was lower, and the frequency of peritoneal EHD was higher in the CRLM + EHD resection group compared to the no resection group. This is unexpected as CRLM + EHD patients with lung metastases are typically selected over patients with peritoneal metastases to undergo curative‐intent resection as lung metastases have been demonstrated to have a lesser impact on survival than liver disease [21]. In larger series, there is data to indicate that 5‐year survival is quite low in patients with peritoneal disease [5, 22, 23]. As discussed below in our study's limitations, this may be a result of peritoneal metastases being low volume and/or incidentally discovered at the time of surgery rather than preoperatively, which our study cannot discern. Additionally, lung metastases, especially those that are low‐volume and subcentimeter in size, may not be coded by physicians as the diagnosis is suspected but not confirmed, or the lung metastases are felt to be clinically inconsequential. These factors may also account for why the 10‐year or more survivors in the CRLM + EHD resection group were comprised of more patients with peritoneal EHD than lung EHD. Regardless, our findings are in line with other recent studies [24, 25] and support consideration of hepatectomy in select CRLM + EHD patients with limited peritoneal disease, and they may reflect changing treatment paradigms as systemic therapy and survival for metastatic CRC continue to improve [26].

As liver‐directed therapies such as TACE and Y90 are often reserved for patients with advanced, unresectable liver disease [27, 28], our findings of improved survival with liver resection over these modalities are expected.

Although our study cannot determine which CRLM + EHD patients should be offered resection as individual tumor burdens are unknown, our results support that hepatectomy for select CRLM + EHD is associated with improved survival. Therefore, as previously shown for liver‐limited disease [29], a liver resection rate of 6% for CRLM + EHD highlights that there may be underutilization of surgery. With appropriate screening, more CRLM + EHD may experience improved survival if offered surgery compared to palliative chemotherapy alone.

As a retrospective, population‐based study, our study has inherent limitations. The CCR‐OSHPD database does not provide information regarding the burden of disease, such as the number of metastatic sites or size of metastases, and it is dependent upon provider billing codes. It is unknown whether EHD was identified before surgery or intraoperatively discovered, and there is likely bias regarding patient selection for surgery. Additionally, we do not describe additional resections of EHD that occurred at the time of hepatectomy and whether all disease was cleared.

Our study is one of the largest cohort studies that we are aware of for patients undergoing hepatectomy in the setting of EHD and reflects real‐world practices. In 2023, the treatment of metastatic CRC continues to evolve with the utilization of newer systemic therapies (including targeted therapies such as anti‐EGFR monoclonal antibodies and immunotherapy) in conjunction with surgical resection after careful selection of patients with favorable biology in the setting of EHD.

## 5 Conclusion

This study demonstrates that in a large population‐based setting, CRLM patients with EHD that undergo hepatectomy can have prolonged survival. As the complex multidisciplinary care of patients with metastatic CRC is evolving, thoughtful patient selection criteria must be developed to determine appropriate surgical candidacy in the context of EHD to optimize survival outcomes in this population.

## Conflicts of Interest

The authors declare no conflicts of interest.

## Synopsis

Liver resection for colorectal liver metastases in the setting of extrahepatic disease is controversial. This large, population‐based study demonstrates that liver resection is associated with prolonged survival and supports surgical intervention with thoughtful patient selection.

## Supporting information

Supporting information.

## Data Availability

The data used for this study are available from the California Cancer Registry, found at http://www.ccrcal.org/retrieve-data/data-for-researchers/how-to-request-ccr-data/.
