# Supplementary material for: Liver Resection With Extrahepatic Disease: A Population‐Based Analysis of Thoughtful Selection
Source: J Surg Oncol. 2024 Oct 28;131(3):443–9. doi: 10.1002/jso.27944 (PMC12044278; doi:10.1002/jso.27944)
Supplement: Supplementary file 1 — Supporting information. [file JSO-131-443-s001.docx]

| **ICD-9-CM**  **Code** | **ICD-9 Diagnosis** | **ICD-10-CM**  **Code** | **ICD-10 Diagnosis** |
| --- | --- | --- | --- |
| 197.7 | Secondary malignant neoplasm of liver | C78.7 | Secondary malignant neoplasm of liver |
| 196.2 | Secondary malignant neoplasm of intra-abdominal lymph nodes | C77.2 | Secondary malignant neoplasm of intra-abdominal lymph nodes |
| 197.0 | Secondary malignant neoplasm of lung | C78.0 | Secondary malignant neoplasm of lung |
| 197.6 | Secondary malignant neoplasm of peritoneum | C78.6 | Secondary malignant neoplasm of peritoneum |
| 197.7 | Secondary malignant neoplasm of liver | C78.7 | Secondary malignant neoplasm of liver |
| 198.3 | Secondary malignant neoplasm of brain | C79.31 | Secondary malignant neoplasm of brain |
| 198.5 | Secondary malignant neoplasm of bone | C79.51-C79.52 | Secondary malignant neoplasm of bone |
| 198.6 | Secondary malignant neoplasm of ovary | C79.6-C79.62 | Secondary malignant neoplasm of ovary |
| 198.7 | Secondary malignant neoplasm of adrenal gland | C79.7-C79.72 | Secondary malignant neoplasm of adrenal gland |

**Supplementary Table 1.** ICD-9-CM and ICD-10-CM diagnoses used to identify patients with colorectal liver metastases and extrahepatic disease.

| **ICD-9-CM**  **Code** | **ICD-9 Diagnosis** | **ICD-10-CM**  **Code** | **ICD-10 Diagnosis** |
| --- | --- | --- | --- |
| 197.7 | Secondary malignant neoplasm of liver | C78.7 | Secondary malignant neoplasm of liver |
| 196.2 | Secondary malignant neoplasm of intra-abdominal lymph nodes | C77.2 | Secondary malignant neoplasm of intra-abdominal lymph nodes |
| 197.0 | Secondary malignant neoplasm of lung | C78.0 | Secondary malignant neoplasm of lung |
| 197.6 | Secondary malignant neoplasm of peritoneum | C78.6 | Secondary malignant neoplasm of peritoneum |
| 197.7 | Secondary malignant neoplasm of liver | C78.7 | Secondary malignant neoplasm of liver |
| 198.3 | Secondary malignant neoplasm of brain | C79.31 | Secondary malignant neoplasm of brain |
| 198.5 | Secondary malignant neoplasm of bone | C79.51-C79.52 | Secondary malignant neoplasm of bone |
| 198.6 | Secondary malignant neoplasm of ovary | C79.6-C79.62 | Secondary malignant neoplasm of ovary |
| 198.7 | Secondary malignant neoplasm of adrenal gland | C79.7-C79.72 | Secondary malignant neoplasm of adrenal gland |

**Supplementary Table 2.** ICD-9-CM and ICD-10-PCS procedure codes used to identify patients who underwent liver resection.

| **ICD-9-CM**  **Code** | **ICD-9 Diagnosis** | **ICD-10-PCS**  **Code** | **ICD-10 Diagnosis** |
| --- | --- | --- | --- |
| 50.22 | Partial Hepatectomy | 0FB00ZZ | Excision of Liver, Open Approach |
|  |  | 0FB04ZZ | Excision of Liver, Percutaneous Endoscopic Approach |
| 50.23 | Open Ablation of Liver Lesion or Tissue | 0F500ZZ | Destruction of Liver, Open Approach |
| 50.25 | Laparoscopic Ablation of Liver Lesion or Tissue | 0F504ZZ | Destruction of Liver, Percutaneous Endoscopic Approach |
| 50.3 | Lobectomy of Liver | 0FT10ZZ | Resection of Right Lobe Liver, Open Approach |
|  |  | 0FT14ZZ | Resection of Right Lobe Liver, Percutaneous Endoscopic Approach |
|  |  | 0FT20ZZ | Resection of Left Lobe Liver, Open Approach |
|  |  | 0FT24ZZ | Resection of Left Lobe Liver, Percutaneous Endoscopic Approach |
| 50.4 | Total Hepatectomy | 0FT00Z | Resection of liver, open approach |
|  |  | 0FT04ZZ | Resection of liver, percutaneous endoscopic approach |
